# Circulating cytokines allow for identification of malignant intraductal papillary mucinous neoplasms of the pancreas

**DOI:** 10.1002/cam4.5051

**Published:** 2022-07-24

**Authors:** Ning Pu, Qiangda Chen, Jicheng Zhang, Hanlin Yin, Dansong Wang, Yuan Ji, Shengxiang Rao, Tiantao Kuang, Xuefeng Xu, Wenchuan Wu, Wenhui Lou

**Affiliations:** ^1^ Department of General Surgery Zhongshan Hospital, Fudan University Shanghai China; ^2^ Cancer Center, Zhongshan Hospital Fudan University Shanghai China; ^3^ Department of Pathology Zhongshan Hospital, Fudan University Shanghai China; ^4^ Department of Radiology Zhongshan Hospital, Fudan University Shanghai China

**Keywords:** circulating cytokine score, intraductal papillary mucinous neoplasm, malignant transformation, nomogram, serum biomarker

## Abstract

**Background:**

Intraductal papillary mucinous neoplasms (IPMNs) are the precursor lesions of pancreatic cancers, requiring active surgical intervention during cancer development. However, the current criteria for predicting malignant IPMNs remain challenging and limited. Hence, this study aimed to assess the discriminatory performance of circulating cytokines, including TNF‐α, IL‐2R, IL‐6, and IL‐8, then build a novel predictive model to improve the diagnostic accuracy.

**Method:**

A total of 131 retrospective (from March 2016 to December 2019) and 53 prospective (from March 2020 to January 2021) patients who were histologically confirmed as IPMNs were consecutively collected and analyzed.

**Result:**

The circulating levels of TNF‐α, IL‐2R, IL‐6, and IL‐8 were significantly elevated in malignant IPMNs, and were verified as independent factors for malignant IPMNs (*p* < 0.05). Then, a novel score, the circulating cytokine score (CCS), was calculated and demonstrated as an independent predictive indicator with a higher area under the curve (AUC) than each cytokine alone (*p* < 0.001). Besides the CCS, two high‐risk stigmata features, the presence of solid component (PSC), and main pancreatic duct (MPD) dilation ≥10 mm were also demonstrated as independent indicators for predicting malignant IPMNs. Finally, a novel nomogram incorporating the CCS and these two high‐risk stigmata features presented a remarkable diagnostic performance, both in the training and validation cohorts with AUCs of 0.928 and 0.873, respectively.

**Conclusion:**

The CCS can be considered a novel independent predictive indicator for malignant IPMNs. Additionally, the formulated nomogram model integrating the CCS, PSC, and MPD ≥10 mm can be a valuable and promising tool for predicting the malignant transformation of IPMNs during long‐term follow‐ups to assist in timely and accurate surgical decisions.

## Graphical abstract

The serum TNF‐, IL‐2R, IL‐6, and IL‐8 in malignant IPMNs are significantly higher than benign ones. Circulating cytokine score (CCS) was calculated and proved as an independent predictor.A novel nomogram incorporating CCS showed a remarkable diagnostic performance for distinguishing malignant IPMNs.

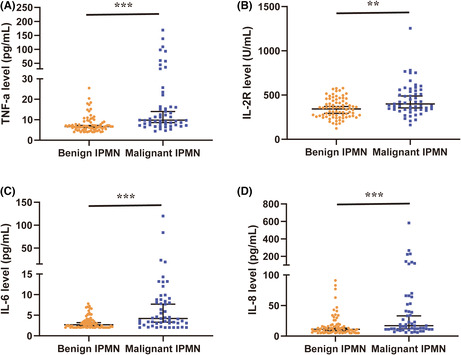

## 1 INTRODUCTION

Pancreatic cancer is one of the most lethal malignancies with a 5‐year survival rate under 10%.^1^ The difficulty in early diagnosis remains one of the pivotal issues for its poor prognosis, and, when patients are diagnosed with pancreatic cancer, only less than 20% can be candidates for curative resection.^2^, ^3^ Thus, the overall prognosis can be enhanced by the early identification of precancerous lesions that might progress to malignancy.

Intraductal papillary mucinous neoplasms (IPMNs) are precancerous lesions. Due to the wide use and development of high‐resolution abdominal imaging, the incidental identification of IPMNs has markedly increased.^4^ These cystic IPMN lesions can gradually progress from low‐grade dysplasia (LGD), intermediate‐grade dysplasia (IGD), and high‐grade dysplasia (HGD) to invasive carcinoma over time, and might account for 20–30% of pancreatic cancers.^5^ Therefore, patients with malignant IPMNs and HGD and invasive carcinoma are ideal surgical targets, whereas those with benign IPMNs, including LGD and IGD, need to be conservatively managed because only a few neoplasms will progress to cancer during the patient's lifetime. However, according to the current World Gastroenterology Organization (WGO) Practice Guideline, IPMNs have just been divided into LGD and HGD.^6^ As reported previously, 10%–15% of branch‐duct type IPMNs (BD‐IPMNs) and 60% of main‐duct type IPMNs (MD‐IPMNs) can become malignant lesions after surgical resection.^7^, ^8^ Thus, early and accurate preoperative discrimination of malignant IPMNs can enhance the overall prognosis and quality of life of patients. The International Consensus Guideline (ICG)^9^ and European guideline^10^ for IPMN management comprehensively evaluated the radiological characteristics, clinical symptoms, and carbohydrate antigen (Ca)19–9 to distinguish benign and malignant IPMNs during follow‐up surveillance. These guidelines recommend that the highly suspected malignant lesion should be surgically resected, otherwise it should be managed by follow‐ups to avoid a potentially life‐threatening surgery. Moreover, Büchler et al.^11^ have explored the timeliness of resection for IPMNs and found that the radiological criteria for malignant conditions were detected with 13.9%, 34.5%, and 88.6% in the too early, timely, and too late groups, respectively. Hence, timely curative resection remains difficult for the preoperative evaluation of IPMNs based on the current criteria. Furthermore, Salvia et al.^12^ have shown that the dynamic variables of worrisome features and high‐risk stigmata are independently correlated with HGD in BD‐IPMNs, which might indicate changes in internal biology and dynamics from IPMNs to cancers. Although the preoperative diagnosis of IPMN is not difficult, the exact time for surgery during long‐term follow‐ups remains unknown and lacks sensitivity. Therefore, more reliable and accurate biomarkers reflecting innate tumor biological features are urgent to assist in the early identification of IPMNs' malignant transformation.

Increasing circulating biomarkers have been identified for malignant IPMNs. For example, MUC5AC in circulating extracellular vesicles has been identified as a potential and meaningful protein for malignant IPMNs.^13^ Thus, blood testing can be a promising, beneficial, and convenient method to discriminate malignant IPMNs for optimal clinical decisions. Additionally, inflammatory markers reflecting the immune status are diagnostic and prognostic predictors in various cancers.^14^, ^15^ IPMNs are also accompanied by inflammation. Accordingly, several inflammatory markers, such as the C‐reactive protein to albumin ratio^16^ and neutrophil‐lymphocyte ratio,^17^ are significantly related to malignant IPMNs. Besides, inflammatory cytokines, including circulating interleukin (IL)‐2R, IL‐6, IL‐8, and tumor necrosis factor‐α (TNF‐α), are significantly increased in many malignancies, such as pancreatic cancer, and are associated with poor prognosis, which might partly reveal the tumor proinflammatory microenvironment and the malignant transformation process.^18^, ^19^, ^20^, ^21^, ^22^, ^23^ However, the discriminatory performance of these four circulating cytokines in predicting malignant IPMNs remains unknown.

Therefore, this study aimed to identify the discriminatory performance of circulating TNF‐α, IL‐2R, IL‐6, and IL‐8 for malignant IPMNs, and formulate a novel circulating cytokine score (CCS) for intensified prediction of malignant IPMNs. Meanwhile, a novel diagnostic nomogram with superior discriminatory power including the CCS and imaging features was further established.

## 2 MATERIAL AND METHODS

### 2.1 Patients

Between March 2016 and December 2019, 152 consecutive patients preoperatively diagnosed and histologically confirmed as IPMNs of any subtype (e.g., branch duct, main duct, or mixed type) were retrospectively enrolled after radical resection at the Zhongshan Hospital, Fudan University. Patients did not have transplantations; human immunodeficiency virus infections; autoimmune diseases; treatments with immunosuppressive agents; and personal history of malignancy. Among these patients, 131 consecutive patients were finally identified and analyzed as a training cohort under the following criteria: complete general and surgical details; preoperative magnetic resonance imaging (MRI) or computed tomography (CT) examinations; and preoperative blood tumor markers and cytokines examinations within 7 days [e.g., Ca19‐9, carcinoembryonic antigen (CEA), TNF‐α, IL‐2R, IL‐6, and IL‐8]. Twenty‐one patients were excluded due to a lack of preoperative circulating cytokine examinations.

Then, another cohort was designed to recruit IPMN patients for validation. The primary endpoint of this study was the sensitivity of the CCS in assessing the malignant transformation of IPMNs. The secondary endpoints included the specificity, positive predictive value (PPV), and negative predictive value (NPV) of the CCS. The validation cohort size was determined using the Power Analysis & Sample Size (PASS) 15 software as follows: the sensitivity of the CCS was 88.7% in the training cohort and was estimated that 47 patients should be analyzed to obtain the sensitivity based on the 95% confidence interval (95% CI) and 10% tolerance error. Finally, 53 consecutive patients with a preoperative diagnosis of IPMNs from March 2020 to January 2021 at the Zhongshan Hospital, Fudan University were prospectively enrolled as the validation cohort. All patients were prospectively examined for these circulating cytokines and pathologically confirmed after radical resection. All surgeries followed the Fukuoka standards.^9^ This study was conducted according to the ethical policies and procedures approved by the Ethics Committee of the Zhongshan Hospital, Fudan University, and the protocol was registered in Research Registry (researchregistry7046).

### 2.2 Clinicopathological data analysis

According to the Fukuoka international consensus^9^ and clinical experiences, high‐risk stigmata with the presence of solid component (PSC), main pancreatic duct (MPD) dilation ≥10 mm and obstructive jaundice, and worrisome features, including MPD = 5–9 mm, cyst size more than 3 cm, thickened enhancing cyst walls, pancreatitis, and lymphadenopathy, were evaluated. All preoperative CT or MRI imaging was assessed by two independent experienced radiologists who knew the pathological diagnosis but were blind to the clinical information. A serum total bilirubin >1.5 mg/dL was defined as obstructive jaundice for lesions located on the pancreatic head, while it was assigned as absent for lesions other than the pancreatic head. Pancreatitis was diagnosed with two or more of the following indications: (1) abdominal pain suggestive of pancreatitis; (2) serum amylase or lipase levels greater than three times the upper normal value; (3) typical imaging manifestations. Meanwhile, characteristics such as local compression, upper abdominal pain, jaundice, pancreatitis history, dyspepsia, surgical procedure, tumor location, IPMN subtype, the elevation of serum level of Ca19‐9 (≥37 U/mL) and CEA (>5 ng/mL) were assessed according to the international consensus. The serum levels of Ca19‐9, CEA, TNF‐α, IL‐2R, IL‐6, and IL‐8 were determined by the Laboratory Medicine, Zhongshan Hospital, Fudan University. The concentrations of TNF‐α, IL‐2R, IL‐6, and IL‐8 were detected by chemiluminescent immunoassay (SIEMENS Healthineers).

All surgical specimens were evaluated by an experienced hepatic‐biliary‐pancreatic pathologist. According to the WGO Practice Guideline and the international consensus for IPMNs, benign IPMNs included LGD and IGD, and malignant IPMNs included HGD and associated invasive carcinomas.^6^

### 2.3 Statistical analyses

The SPSS 21.0 (IBM) and R software version 4.0.2 (Bell Laboratories) were used for statistical analyses. Continuous variables were depicted by the medians and inter‐quartile ranges (IQRs) and analyzed using the Student's *t*‐test or Mann–Whitney *U* test. The differences in categorical variables between benign and malignant IPMNs were analyzed by the Pearson χ^2^ test or Fisher's exact test. The levels of circulating cytokines were depicted with scatter diagrams plotted by GraphPad Prism 8 (GraphPad Software). The optimal cut‐off values of circulating cytokines were determined by the areas under the receiver operating characteristic (ROC) curves (AUCs) and their Youden index. The sensitivity, specificity, PPV, and NPV were calculated for every potential model. Univariable and multivariable Logistic regression analyses were applied for evaluating the independent risk factors for malignant IPMNs. In 2‐tailed analyses, statistical significance was characterized by a *p‐*value <0.05.

The diagnostic nomogram based on the PSC, MPD, and CCS was formulated by R software. Its diagnostic power was further assessed by calibration curves, concordance index (C‐index), decision curve analysis (DCA), and clinical impact curve (CIC) as previously described.^24^, ^25^

## 3 RESULTS

### 3.1 Demographic and clinical features of patients

According to the retrospective criteria, 131 IPMN patients after surgical resection were enrolled in the training cohort. In this cohort, 53 (40.5%) patients had malignant IPMNs, including 30 patients with invasive carcinoma. The median age was 65.0 (IQR, 60.0–71.0) years, and the median diameter of cyst and MPD was 2.5 (IQR, 2.0–3.7) cm and 5.0 (IQR, 3.3–7.8) mm, respectively. High‐risk stigmata and worrisome features are summarized in Table S1.

Following the prospective inclusion and exclusion criteria, 53 patients with IPMNs were included in the validation cohort. In this cohort, 21 (39.6%) patients had malignant IPMNs, including 11 patients with invasive carcinoma. The median age was 66.0 (IQR, 62.0–71.0) years, and the median diameter of cyst and MPD was 2.5 (IQR, 1.7–4.0) cm and 6.2 (IQR, 3.4–9.3) mm, respectively. Meanwhile, no significant differences in clinical, pathologic, and radiographic characteristics were detected between the validation and training cohorts (Table S1).

### 3.2 Discriminatory performance of circulating cytokines, imaging, and clinical features in malignant IPMNs

**FIGURE 1:**
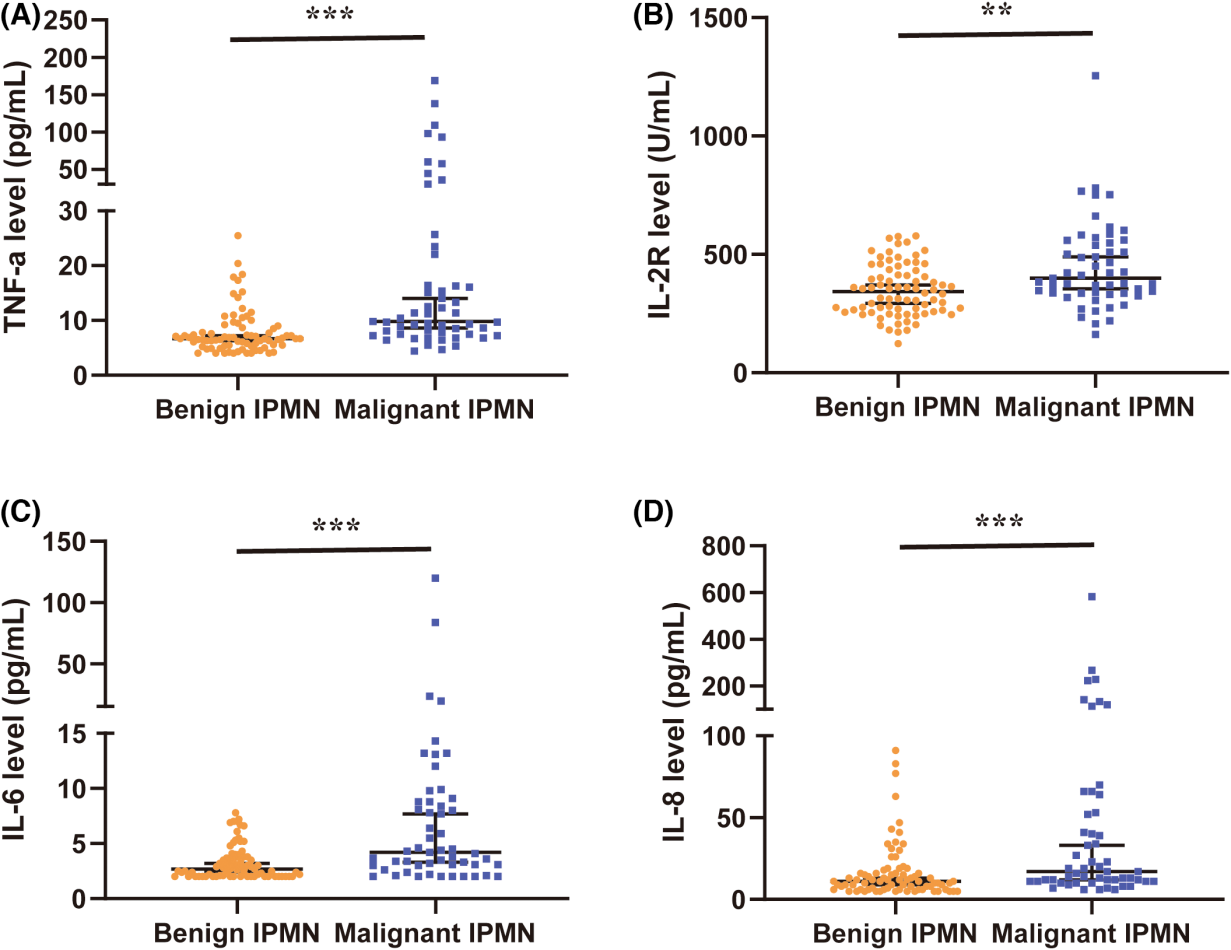
Serum concentrations of four cytokines in IPMN patients from the training cohort. The comparison of serum (A) TNF‐α, (B) IL‐2R, (C) IL‐6, and (D) IL‐8 levels in benign and malignant IPMNs, respectively. The longer horizontal bars represent the median, and the shorter represent the 75th and 25th percentiles. IPMNs, intraductal papillary mucinous neoplasms. **p < 0.01; ***p < 0.001.

The discriminatory abilities of clinical, pathologic, and radiographic features for malignant IPMNs in the training cohort are presented in Table 1. The results showed that symptoms of local compression (*p* = 0.044), jaundice (*p* = 0.044), surgery type of Whipple (*p* = 0.003), main‐duct IPMN subtype (*p* = 0.004), larger cyst diameter (*p* = 0.041), larger MPD dilation (*p* = 0.001), and higher serum Ca19‐9 levels (*p* < 0.001) were more common in malignant IPMN patients. Considering the high‐risk stigmata and worrisome features, obstructive jaundice (*p* = 0.044), PSC (*p* < 0.001), MPD dilation ≥10 mm (*p* = 0.002), and lymphadenopathy (*p* = 0.029) were significantly different in malignant IPMNs. Meanwhile, the circulating cytokines TNF‐α, IL‐2R, IL‐6, and IL‐8 were remarkably higher in malignant IPMNs and were positively correlated (all *p* ≤ 0.001, Figure 1). However, other general clinical characteristics and worrisome features did not present significant differences (Table 1).

**TABLE 1:** Demographic and clinical characteristics of patients with malignant IPMNs in the training cohort

Variables	Benign IPMN (*n* = 78)	Malignant IPMN (*n* = 53)	*p* value
Age, median (IQR)	64.0 (59.0–69.3)	68.0 (60.0–72.5)	0.096
Gender, *n* (%)			0.241
Female	36 (46.2)	19 (35.8)	
Male	42 (53.8)	34 (64.2)	
Clinical, *n* (%)			
Symptoms	34 (43.6)	28 (52.8)	0.298
Local compression	2 (2.6)	7 (13.2)	**0.044**
Upper abdominal pain	22 (28.2)	19 (35.8)	0.354
Jaundice	2 (2.6)	7 (13.2)	**0.044**
Pancreatitis history	7 (9.0)	6 (11.3)	0.659
Dyspepsia	22 (28.2)	21 (39.6)	0.172
Surgery, *n* (%)			**0.003**
cWhipple	21 (26.9)	28 (52.8)	
ppWhipple	9 (11.5)	4 (7.5)	
Distal pancreatectomy spleen preserving	6 (7.7)	1 (1.9)	
Distal pancreatectomy with splenectomy	33 (42.3)	14 (26.4)	
Local resection	3 (3.8)	0	
Enucleation	4 (5.1)	0	
Total resection	2 (2.6)	6 (11.3)	
Location, *n* (%)			0.262
Head	36 (46.2)	33 (62.3)	
Body	29 (37.2)	13 (24.5)	
Tail	12 (15.4)	6 (11.3)	
Overlaps	1 (1.3)	1 (1.9)	
Subtype, *n* (%)			**0.004**
Branch‐duct IPMN	48 (61.5)	17 (32.1)	
Main‐duct IPMN	6 (7.7)	8 (15.1)	
Mixed‐type IPMN	24 (30.8)	28 (52.8)	
Cyst diameter (cm), median (IQR)	2.5 (1.6–3.5)	3.0 (2.0–4.4)	**0.041**
Main duct diameter (mm), median (IQR)	4.3 (3.1–6.5)	6.0 (4.0–10.9)	**0.001**
High‐risk stigmata, *n* (%)			
Obstructive jaundice	2 (2.6)	7 (13.2)	**0.044**
Solid component	2 (2.6)	24 (45.3)	**<0.001**
MPD dilation ≥10 mm	8 (10.3)	17 (32.1)	**0.002**
Worrisome features, *n* (%)			
Cyst size >3 cm	32 (41.0)	28 (52.8)	0.183
Pancreatitis	7 (9.0)	6 (11.3)	0.659
Thickened enhancing cyst walls	12 (15.4)	13 (24.5)	0.191
MPD 5–9 mm	22 (28.2)	19 (35.8)	0.354
Lymphadenopathy	8 (10.3)	13 (24.5)	**0.029**
Tumor biomarkers, *n* (%)			
Ca19‐9 ≥ 37 U/mL	9 (11.5)	22 (41.5)	**<0.001**
CEA >5 ng/mL	8 (10.3)	9 (17.0)	0.261
Circulating cytokines, median (IQR)			
TNF‐α, pg/mL	6.7 (5.4–8.8)	9.8 (7.5–19.3)	**<0.001**
IL‐2R, U/mL	343.5 (262.5–432.8)	399 (335.5–553.5)	**0.001**
IL‐6, pg/mL	2.7 (2.0–3.9)	4.2 (3.0–8.8)	**<0.001**
IL‐8, pg/mL	11.0 (6.0–16.5)	17.0 (11.0–52.5)	**<0.001**
Pathologic grade, *n* (%)			—
Low‐grade dysplasia	27 (34.6)	—	
Intermediate‐grade dysplasia	51 (65.2)	—	
High‐grade dysplasia	—	23 (43.4)	
Invasive carcinoma	—	30 (56.6)	

The *p* values in bold represent statistical significance.

In the validation cohort, high‐risk stigmata of PSC and MPD dilation ≥10 mm, and the worrisome feature of thickened enhancing cyst walls were also significantly related to the malignant transformation of IPMNs (all *p* < 0.05, Table S2). Similarly, compared to benign IPMNs, all four circulating cytokines (TNF‐α, IL‐2R, IL‐6, and IL‐8) were validated as remarkably increased in malignant IPMNs (Figure S1).

### 3.3 Diagnostic performance of the circulating cytokine score (CCS) for predicting malignant IPMNs

The univariable and multivariable analyses within the training cohort showed that the four circulating cytokines ‐ IL‐2R (*p* < 0.001, OR = 1.008, 95% CI, 1.004–1.012), IL‐6 (*p* = 0.003, OR = 1.419, 95% CI, 1.130–1.781), IL‐8 (*p* = 0.008, OR = 1.020, 95% CI, 1.005–1.035), and TNF‐α (*p* = 0.013, OR = 1.115, 95% CI:1.023–1.215) ‐ were independent risk indicators for malignant IPMNs (Table 2). Therefore, according to the regression coefficient, the CCS was calculated as follows: (0.109◊TNF‐α) + (0.008◊IL‐2R) + (0.35◊IL‐6) + (0.02◊IL‐8) ‐ 6.439.

**TABLE 2:** The circulating cytokine model was formulated by univariate and multivariate Logistic regression analysis in the training cohort

Variables	Univariable *p* value	Multivariate *p* value	*β*	OR	95% CI
TNF‐α	0.001	0.013	0.109	1.115	1.023–1.215
IL‐2R	<0.001	<0.001	0.008	1.008	1.004–1.012
IL‐6	<0.001	0.003	0.350	1.419	1.130–1.781
IL‐8	0.005	0.008	0.020	1.020	1.005–1.035

*Note*: Circulating cytokine score (TNF‐α&IL‐2R&IL‐6&IL‐8): Logit (P) = −6.439 + 0.109*TNF‐α + 0.008*IL‐2R + 0.35*IL‐6 + 0.02*IL‐8.

**FIGURE 2:**
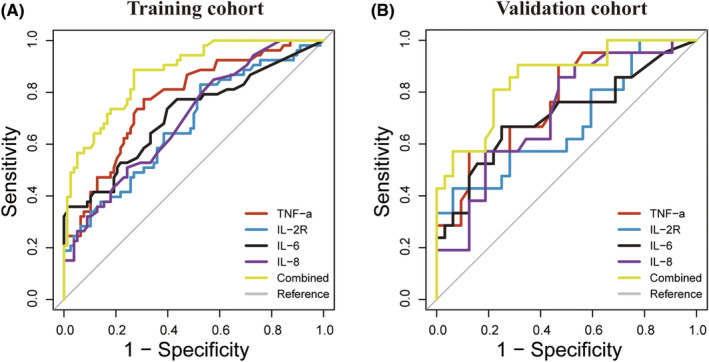
The predictive power of each model was represented by the AUCs. Comparison of the circulating cytokine score (CCS) with TNF‐α, IL‐2R, IL‐6, and IL‐8 alone for predicting malignant IPMNs in the (A) training and (B) validation cohorts. AUC, area under the curve; IPMNs, intraductal papillary mucinous neoplasms.

According to the ROC curves, the most optimal cut‐off values for TNF‐α, IL‐2R, IL‐6, and IL‐8 were 7.45 pg/mL, 317 U/mL, 7.45 pg/mL, and 9.5 pg/mL in the training cohort, respectively. The diagnostic power, including the sensitivity, specificity, PPV, and NPV, were further calculated for these circulating cytokines (Table 3). Additionally, the optimal threshold value for the CCS was −0.72, and the AUC of the CCS (0.874, 95% CI, 0.817–0.932) was significantly larger compared to any single cytokine with sensitivity, specificity, PPV, and NPV of 0.887, 0.731, 0.681 and 0.905, respectively. These results demonstrated the superior discriminatory abilities of the CCS for malignant IPMNs (Figure 2A). Similarly, compared to the individual cytokines, the CCS also provided the highest AUC in the validation cohort (0.853, 95% CI, 0.748–0.957) with sensitivity, specificity, PPV, and NPV of 0.905, 0.687, 0.655, and 0.917, respectively (Figure 2B, Table S3).

**TABLE 3:** Discriminatory performance of TNF‐α, IL‐2R, IL‐6, IL‐8 and their combined model for detecting malignant IPMNs in the training cohort

Variables	AUC (95% CI)	Cutoff level	Sensitivity	Specificity	PPV	NPV
TNF‐α	0.774 (0.693–0.854)	7.45	0.774	0.692	0.631	0.818
IL‐2R	0.674 (0.580–0.768)	317	0.83	0.474	0.518	0.804
IL‐6	0.711 (0.618–0.804)	7.45	0.358	0.987	0.950	0.694
IL‐8	0.696 (0.606–0.786)	9.5	0.849	0.423	0.500	0.805
TNF‐α&IL‐2R&IL‐6&IL‐8	0.874 (0.817–0.932)	−0.72	0.887	0.731	0.681	0.905

*Note*: Circulating cytokine score (TNF‐α&IL‐2R&IL‐6&IL‐8): Logit (P) = −6.439 + 0.109*TNF‐α + 0.008*IL‐2R + 0.35*IL‐6 + 0.02*IL‐8.

Abbreviations: AUC, area under the receiver‐operating‐characteristic curve; CI, confidence interval; NPV, negative predictive value; PPV, positive predictive value.

### 3.4 Independent predictive indicators for malignant IPMNs

To identify the independent predictive factors for malignant IPMNs, the univariable analysis was used and showed that local compression (*p* = 0.033), obstructive jaundice (*p* = 0.033), PSC (*p* < 0.001), MPD dilation ≥10 mm (*p* = 0.003), lymphadenopathy (*p* = 0.033), Ca19‐9 ≥ 37 U/mL (*p* < 0.001), and CCS (*p* < 0.001) were significant predictors for malignant IPMNs in the training cohort. Additionally, the multivariable analysis demonstrated that the PSC (*p* < 0.001, OR = 108.340, 95% CI, 7.597–1544.979), MPD dilation ≥10 mm (*p* = 0.014, OR = 16.989, 95% CI, 1.794–160.849), and CCS (*p* < 0.001, OR = 84.386, 95% CI, 84.386) were independent predictive indicators for malignant IPMNs (Table 4).

**TABLE 4:** Independent risk factors associated with malignant IPMNs by univariate and multivariate Logistic regression analysis in the training cohort

Variables	Univariable *p* value	Multivariate *p* value	OR	95% CI
Age (≥ 65 years)	0.215	—		
Gender	0.242	—		
Local compression	**0.033**	0.926	1.142	0.068–19.189
Surgery	0.156	—		
Subtype	0.206	—		
Cyst diameter (cm)	0.095	—		
Obstructive jaundice	**0.033**	0.926	1.142	0.068–19.189
Solid component	**<0.001**	**<0.001**	108.340	7.597–1544.979
MPD dilation (≥10 mm)	**0.003**	**0.014**	16.989	1.794–160.849
Lymphadenopathy	**0.033**	0.755	1.267	0.286–5.614
Ca19‐9 (≥37 U/mL)	**<0.001**	0.294	1.947	0.561–6.763
Circulating cytokine score	**<0.001**	**<0.001**	84.386	9.877–721.002

The *p* values in bold represent statistical significance.

For further external validation, the univariable analysis showed that the PSC (*p* = 0.008), MPD dilation ≥10 mm (*p* = 0.005), thickened enhancing cyst walls (*p* = 0.018), Ca19‐9 ≥ 37 U/mL (*p* = 0.039), and CCS (*p* < 0.001) were potential predictive indicators for the presence of malignant IPMNs in the validation cohort. In the subsequent multivariate analysis, the PSC (*p* = 0.027, OR = 20.932, 95% CI: 1.416–309.493), MPD dilation ≥10 mm (*p* = 0.004, OR = 76.417, 95% CI, 3.839–1520.945), thickened enhancing cyst walls (*p* = 0.048, OR = 10.887, 95% CI, 1.026–115.563), Ca19‐9 ≥ 37 U/mL (*p* = 0.025, OR = 16.216, 95% CI, 1.408–186.713), and CCS (*p* = 0.010, OR = 20.188, 95% CI, 2.053–198.497) were independent predictors for malignant IPMNs. Altoghther, these results demonstrated the robust predictive role of the PSC, MPD dilation ≥10 mm, and CCS (Table S4).

### 3.5 Establishment of a diagnostic nomogram incorporating the CCS and imaging features

**FIGURE 3:**
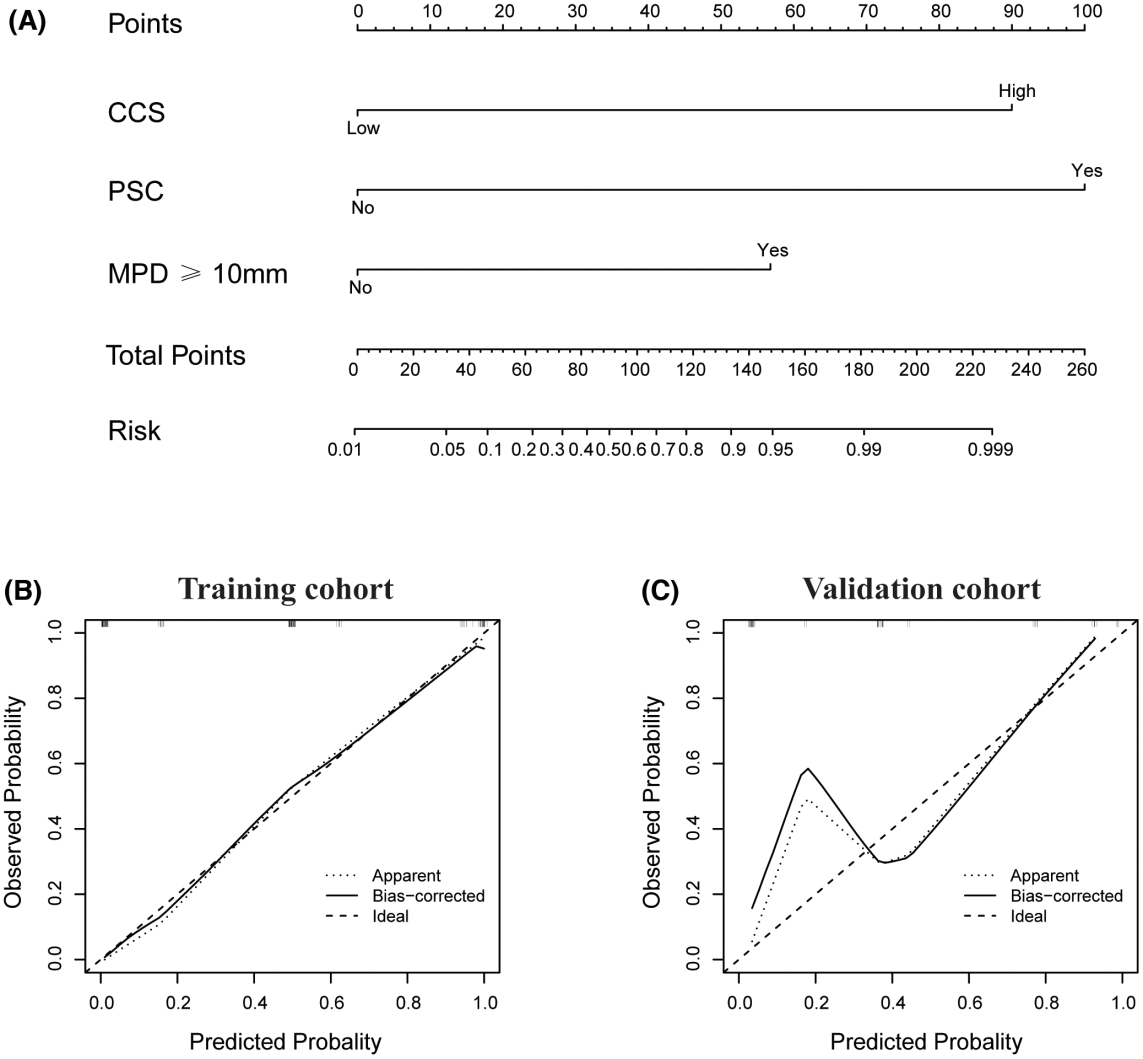
Establishment and validation of a novel nomogram model. (A) A novel nomogram integrating the circulating cytokine score (CCS), presence of solid component (PSC), and main pancreatic dilation (MPD) ≥ 10 mm was established to predict the malignant potential of IPMNs. Calibration curves for evaluating the observed and nomogram‐predicted probability of malignancies in the (B) training and (C) validation cohorts. IPMNs, Intraductal papillary mucinous neoplasms.

Based on the univariable and multivariable analysis results in the training and validation cohorts, a novel diagnostic nomogram incorporating the CCS, PSC, and MPD ≥10 mm was formulated to predict the malignant potential of IPMNs (Figure 3A). The C‐index of this novel predictive nomogram reached 0.928 (95% CI, 0.888–0.969), remarkably higher than each indicator alone [CCS, 0.809 (95% CI, 0.732–0.886); PSC, 0.714 (95% CI, 0.618–0.810); MPD ≥10 mm, 0.609 (95% CI, 0.508–0.710)]. Furthermore, a perfect consistency was detected between the nomogram‐predicted and actual‐observed malignant IPMNs by the calibration curves in the training cohort (Figure 3B).

Then, the predictive accuracy of the formulated nomogram for predicting malignant IPMNs in the training cohort was further demonstrated using DCA and CIC with a prominent evaluation for clinical net benefit. Compared to each indicator alone, the nomogram presented a wider range of high‐risk thresholds with superior net benefit, indicating a remarkable improvement in predicting the probability of malignancy development (Figures S2A and S3A).

### 3.6 External validation of the nomogram

According to the formulated nomogram, the relevant total points for each patient were further calculated for the validation cohort. Compared to each indicator alone (CCS, 0.781 [95% CI, 0.653–0.908]; PSC, 0.667 [95% CI, 0.511–0.824]; MPD ≥10 mm, 0.683 [95% CI, 0.527–0.839]), the C‐index of the nomogram model was significantly higher (0.873 [95% CI, 0.773–0.972]). Additionally, the calibration curves presented good agreement in the actual‐observed and nomogram‐predicted values in the validation cohort (Figure 3C).

Considering the predictive accuracy and clinical net benefit for predicting malignant IPMNs, the formulated nomogram was further verified as superior compared to each indicator alone by the DCA and CIC in the validation cohort (Figures S2B and S3B).

## 4 DISCUSSION

Once IPMNs, a known radiologically identifiable precursor of pancreatic cancers, develop into invasive diseases, the prognosis of patients is significantly poorer compared to those without invasion. Therefore, accurately distinguishing benign and malignant IPMNs is crucial to determining the appropriate management of patients. Ideally, surgical resection would be recommended for patients with malignant IPMNs, while those with benign diseases should be conservatively monitored to avoid perioperative morbidity until the disease develops. However, the exact identification of benign or malignant lesions in preoperative clinical practice can be challeging.^26^ Herein, among the 184 patients who experienced resection for IPMNs according to Fukuoka standards, 110 (60%) had LGD or IGD and would benefit more from long‐term follow‐ups. Although the ICG provided a framework for the management of IPMNs and has been validated in many studies, its discriminatory performance for malignant IPMNs remains limited.^27^, ^28^, ^29^ Hence, more predictors have been explored to increase the discriminatory power.

Recently, the incorporation of cyst fluid markers into clinical and radiographic models has improved the diagnostic discrimination of malignant IPMNs.^30^, ^31^, ^32^ For example, Efishat et al.^30^ have incorporated four cyst fluid markers (CA72‐4, sFASL, MMP9, and IL‐4) into a previously established nomogram, and significantly improved its discrimination with C‐indices of 0.84 and 0.83 in two separate cohorts. However, the cyst fluid was mostly collected invasively at the time of preoperative endoscopy or surgical resection, limiting its clinical value for predicting malignant IPMNs. Therefore, in the present study, we focused on a more promising, non‐invasive, convenient, and economical blood testing, and found that four inflammatory markers, TNF‐α, IL‐2R, IL‐6, and IL‐8, were significantly correlated to malignant IPMNs and provided better performance for the clinical decision process.

The proinflammatory cytokine TNF‐α exerts an essential role within the host's immune response,^33^ whereas many studies have recently reported its dual role in the initiation and progression of cancers.^34^ Additionally, TNF‐α promoter gene polymorphisms are significantly correlated to the development of IPMNs and pancreatic cancer and might be produced and released into the microenvironment and circulation during the development and progression.^35^ IL‐2, another proinflammatory cytokine, can activate the proliferation of T cells and trigger a robust immune response and is difficult and unstable to measure due to its very short half‐life. In contrast, serum IL‐2R is suitable and stable to quantify the number of T cells activated by IL‐2 because of its comparatively longer half‐life.^36^ Additionally, increased levels of serum IL‐2R have been found in patients with various cancers, including pancreatic cancer, and might reflect the stage of diseases.^36^, ^37^ Thus, the determination of circulating IL‐2R levels might provide supplementary information for predicting the malignancy of IPMNs. Moreover, IL‐6 and IL‐8 play a major role in the inflammation of pancreatic cancer, and these two cytokines in the circulation are positively associated with pancreatic cancer progression and predicted poor prognosis.^23^, ^38^, ^39^, ^40^, ^41^ Besides, the concentration of IL‐8 in the cyst fluid of malignant lesions is higher compared to benign IPMNs, which might be a potential predictor for malignant diseases.^42^ In the present study, these four promising circulating cytokines were combined for the first time to evaluate malignant IPMNs. Results showed that the levels of TNF‐α, IL‐2R, IL‐6, and IL‐8 were positively associated with the presence of malignancies. All cytokines were verified as independent predictors for malignant IPMNs after the multivariate analysis. Then, a novel CCS was formulated and validated and presented showing better predictive performance with higher AUC values for detecting the malignant IPMNs in the training and validation cohorts (0.874 and 0.853, respectively).

In addition to the CCS, two other imaging characteristics from the Fukuoka standards were identified as independent predictive indicators for malignant IPMNs, the PSC and MPD ≥10 mm. Both are high‐risk stigmata of IPMNs and have been validated as powerful independent predictors for malignant IPMNs in various studies.^17^, ^43^, ^44^, ^45^ More importantly, our nomogram integrating CCS and high‐risk imaging features presented an outstanding diagnostic performance with a C‐index of 0.928 in the training cohort and 0.873 in the validation cohort, significantly higher than current existed predictive models.

Currently, few studies have established a nomogram based on multiple indicators to predict malignant IPMNs.^44^, ^46^, ^47^, ^48^ For example, Shimizu et al.^46^ have built a predictive nomogram for malignant IPMNs incorporating pancreatic juice cytology, and clinical and imaging data, which provided a prominent diagnostic performance (AUC of 0.903). However, the performance of the nomogram in the validation cohort from three high‐volume centers in Japan was relatively poor (AUC of 0.760), partly due to the difficulties in standardizing the diagnostic criteria for cytology.^49^ Meanwhile, obtaining pancreatic juice is an invasive procedure, which further limited its value in clinical application. In contrast, the measurement of the circulating cytokines used here just needs a small amount of blood, which can be easily obtained preoperatively or during follow‐up. Moreover, all parameters in the nomogram of this study are non‐invasive, convenient, objective, and easily accessible, suggesting that it can be widely used in clinical practice. Furthermore, Jung et al.^50^ have validated a nomogram containing MPD, cyst size, PSC, serum CEA, Ca19‐9 levels, and age with C‐indices of 0.745 for Eastern and 0.856 for Western patients to predict malignant IPMNs, lower than our current model.

However, this study also has some limitations. First, it was a single‐center study with finite enrolled cases. Only patients with histologically confirmed IPMNs were included to develop the model, which might difficult its applications to patients with suspected IPMNs under surveillance. Thus, longitudinal analysis of these circulating cytokines and high‐risk imaging features during surveillance in the future can be used to validate its clinical value. Second, the cut‐off value of the CCS might not be appropriate for other patient cohorts. Thus, further large‐scale, multi‐center, prospective clinical trials and meta‐analyses including various CCS studies are required for validation and to determine the most suitable cut‐off. Third, the circulating cytokine levels in patients with immunological function disorder, synchronous malignant diseases, or even viral infections, can result in false positives. In these cases, the circulating cytokine model would be not applicable. Therefore, we adopted the exclusion criteria mentioned above, and other potential influences need to be further evaluated in clinical practice.

## 5 CONCLUSION

In summary, the serum levels of TNF‐α, IL‐2R, IL‐6, and IL‐8 were significantly higher in patients with malignant IPMNs compared to those with benign IPMNs. The CCS was further verified as an independent predictive indicator for the malignant transformation of IPMNs, as well as two high‐risk stigmata features, the PSC and MPD ≥10 mm. Furthermore, a novel predictive nomogram, integrating the CCS, PSC, and MPD ≥10 mm, was established and validated, and remarkably enhanced the predictive power to discriminate malignant IPMNs from benign lesions. Therefore, it can be a valuable and promising tool for predicting the probability of malignant IPMNs in clinical practice and allow timely and accurate surgical decisions.

## AUTHORS' CONTRIBUTIONS

NP, WW and WL had the idea for and designed the study and had full access to all of the data in the study and take responsibility for the integrity of the data and the accuracy of the data analysis. NP, QC, WW and WL drafted the paper. NP, QC, JZ, HY and WL did the analysis, and all authors critically revised the manuscript for important intellectual content and gave final approval for the version to be published. NP, QC, JZ, HY, DW, YJ, SR, TK, XX, WW and WL collected the data. All authors agree to be accountable for all aspects of the work in ensuring that questions related to the accuracy or integrity of any part of the work are appropriately investigated and resolved.

## FUNDING INFORMATION

This study was funded by the National Natural Science Foundation of China (82103409), China Postdoctoral Science Foundation (2021M690037), Shanghai Sailing Program (21YF1407100), National Key R&D Program of China (2019YFC1315902), Youth Fund of Zhongshan Hospital Fudan University (LCBSHZX003) and Shanghai ShenKang Hospital Development Centre Project (SHDC2020CR2017B).

## CONFLICT OF INTEREST

The authors have no conflict of interest.

## ETHICS STATEMENT

The protocol of this study was conducted according to the ethical policies and procedures approved by the Ethics Committee of Zhongshan Hospital, Fudan University and the protocol was registered in Research Registry (researchregistry7046). All candidate subjects have signed written informed consent.

## Supporting information


Figure S1

Figure S2

Figure S3

Table S1

Table S2

Table S3

Table S4

## Data Availability

The data that support the findings of this study are available on request from the corresponding author.
