# Supplementary figures and images for: Circulating cytokines allow for identification of malignant intraductal papillary mucinous neoplasms of the pancreas
Source: Cancer Med. 2022 Jul 24;12(4):3919–30. doi: 10.1002/cam4.5051 (PMC9972143; doi:10.1002/cam4.5051)

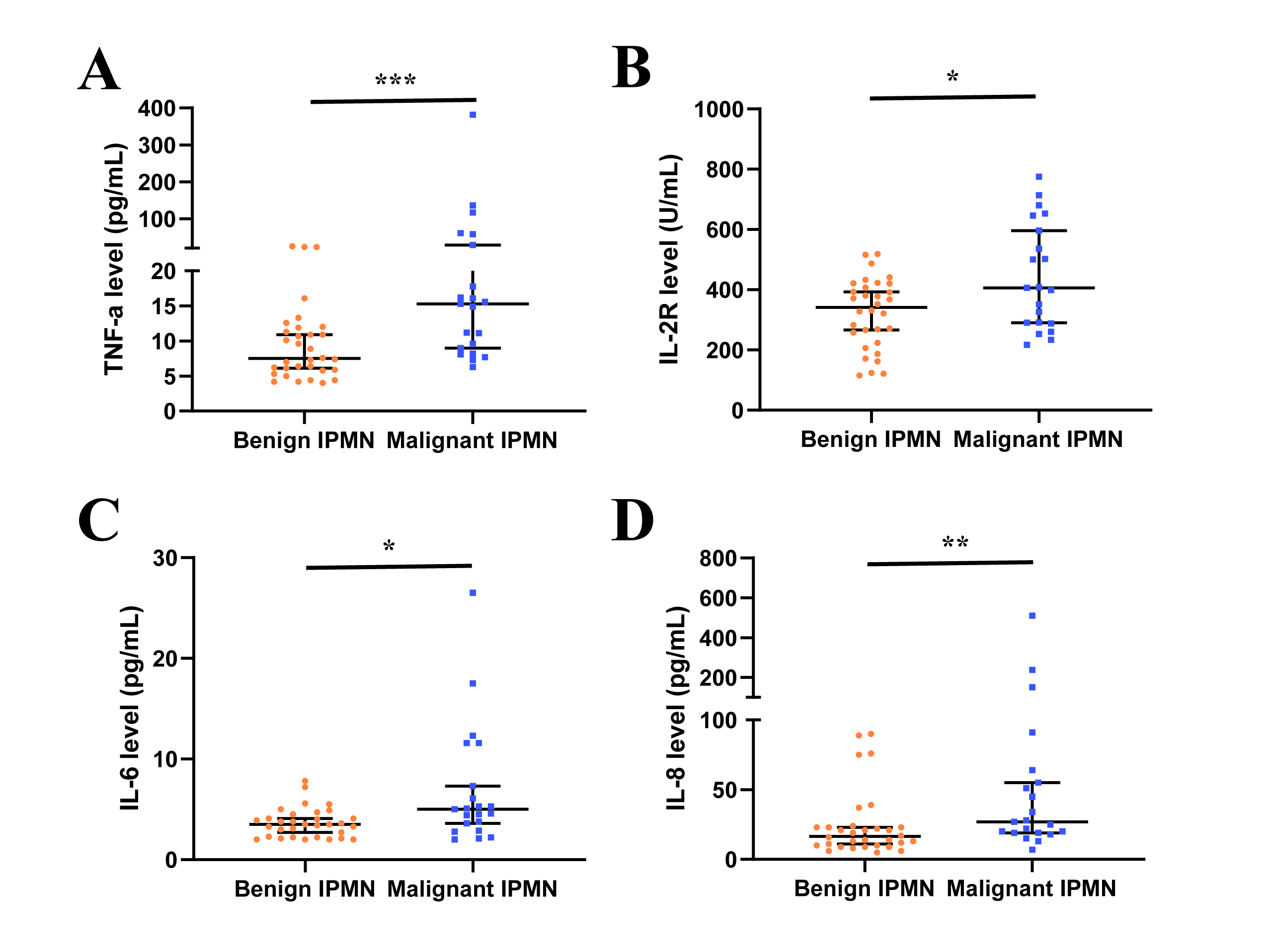

Supplement: Supplementary file 1 — Figure S1 [file CAM4-12-3919-s002.tif]

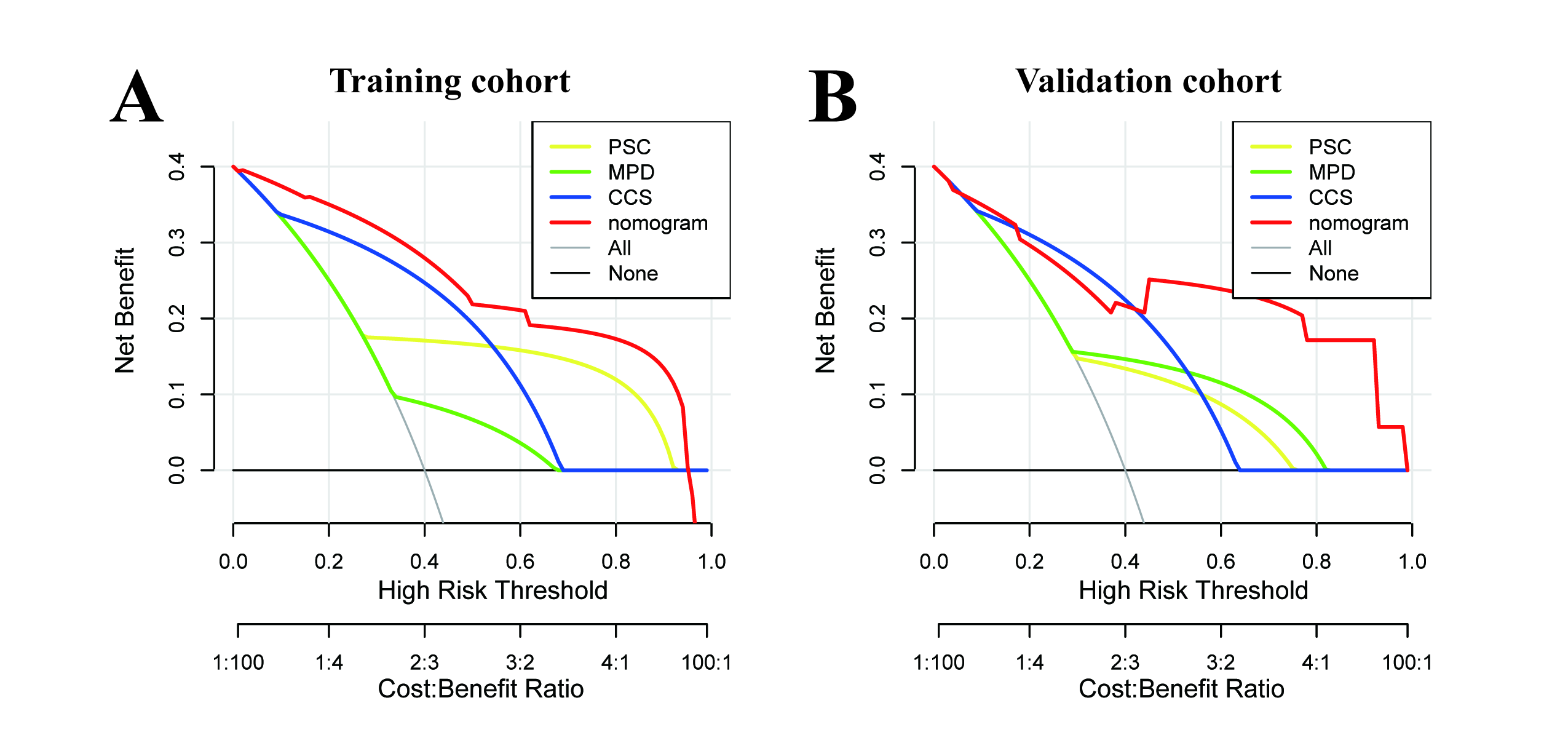

Supplement: Supplementary file 2 — Figure S2 [file CAM4-12-3919-s003.tif]

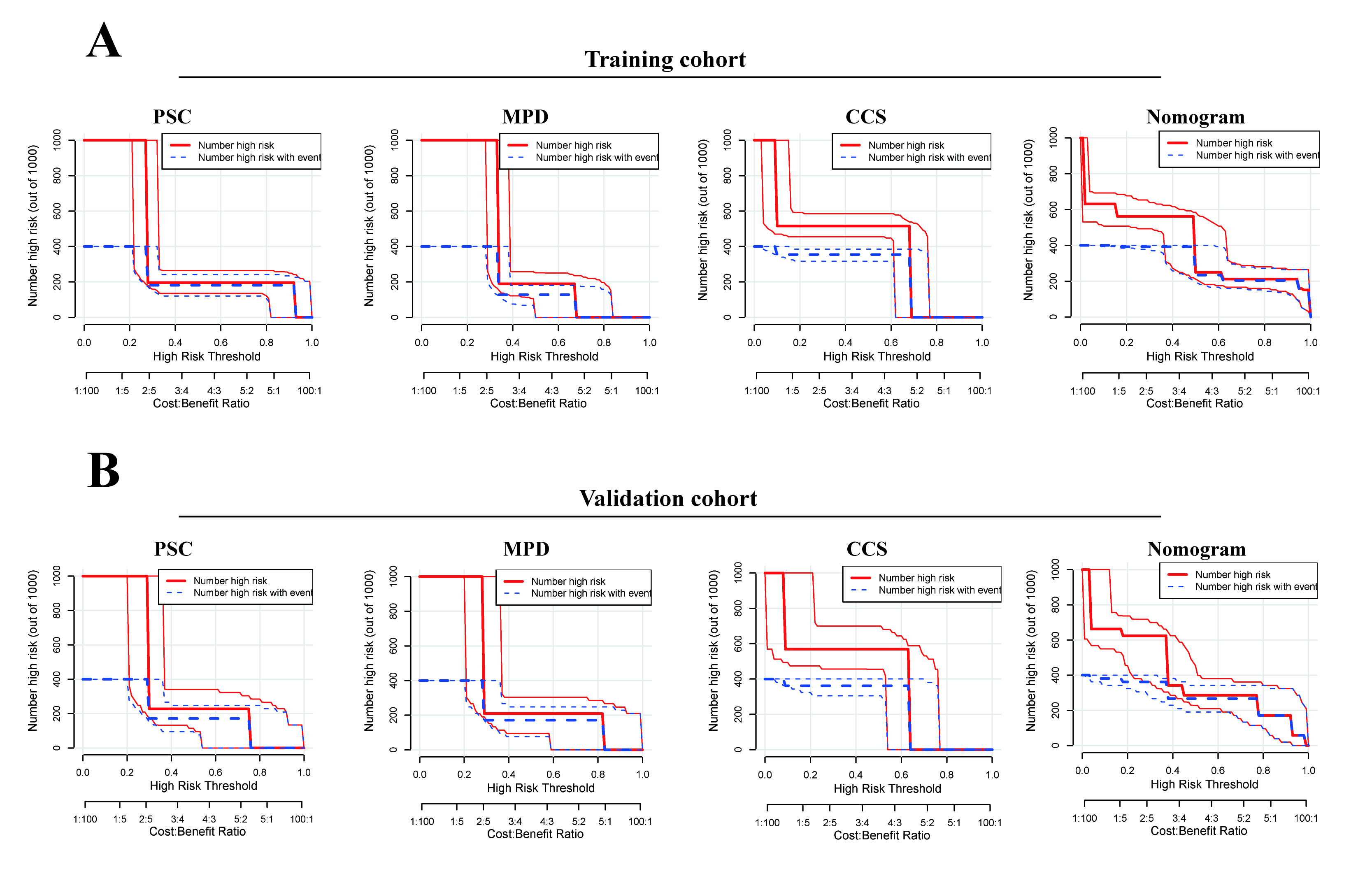

Supplement: Supplementary file 3 — Figure S3 [file CAM4-12-3919-s007.tif]
