# Supplementary material for: Circulating cytokines allow for identification of malignant intraductal papillary mucinous neoplasms of the pancreas
Source: Cancer Med. 2022 Jul 24;12(4):3919–30. doi: 10.1002/cam4.5051 (PMC9972143; doi:10.1002/cam4.5051)
Supplement: Supplementary file 4 — Table S1 [file CAM4-12-3919-s005.docx]

**Supplementary Table 1. The baseline of demographic and clinical characteristics in the training and validation cohort.**

| **Variables** | **Training cohort**  **(n=131)** | **Validation cohort**  **(n=53)** | **P value** |
| --- | --- | --- | --- |
| **Age****, median (IQR)** | 65.0 (60.0-71.0) | 66.0 (62.0-71.0) | 0.273 |
| **Gender, n (%)**  Female  Male | 55 (42.0)  76 (58.0) | 24 (45.3)  29 (54.7) | 0.402 |
| **Clinical, n (%)**  Symptoms  Local compression  Upper abdominal pain  Jaundice  Pancreatitis history  Dyspepsia | 62 (47.3)  9 (6.9)  41 (31.3)  9 (6.9)  13 (9.9)  43 (32.8) | 20 (37.7)  1 (1.9)  14 (26.4)  2 (3.8)  2 (3.8)  10 (18.9) | 0.236  0.322  0.512  0.646  0.279  0.058 |
| **Surgery, n (%)**  cWhipple  ppWhipple  Distal pancreatectomy spleen preserving  Distal pancreatectomy with splenectomy  Local resection  Enucleation  Total resection | 49 (37.4)  13 (9.9)  7 (5.3)  47 (35.9)  3 (2.3)  4 (3.1)  8 (6.1) | 25 (47.2)  0 (0)  1 (1.9)  23 (43.4)  1 (1.9)  0 (0)  3 (5.7) | 0.152 |
| **Location, n (%)**  Head  Body  Tail  Overlaps | 69 (52.7)  42 (32.1)  18 (13.7)  2 (1.5) | 27 (50.9)  18 (34.0)  7 (13.2)  1 (1.9) | 0.992 |
| **Subtype, n (%)**  Branch-duct IPMN  Main-duct IPMN  Mixed-type IPMN | 65 (49.6)  14 (10.7)  52 (39.7) | 18 (34.0)  10 (18.9)  25 (47.2) | 0.106 |
| **Cyst diameter (cm), median (IQR)** | 2.5 (2.0-3.7) | 2.5 (1.7-4.0) | 0.935 |
| **Main duct diameter (mm), median (IQR)** | 5.0 (3.3-7.8) | 6.2 (3.4-9.3) | 0.183 |
| **High-risk stigmata, n (%)**  Obstructive jaundice  Solid component  MPD dilation ≥ 10 mm | 9 (6.9)  26 (19.8)  25 (19.1) | 1 (1.9)  12 (22.6)  11 (20.8) | 0.332  0.672  0.796 |
| **Worrisome features, n (%)**  Cyst size > 3 cm  Pancreatitis  Thickened enhancing cyst walls  MPD 5 - 9 mm  Lymphadenopathy | 60 (45.8)  13 (9.9)  25 (19.1)  41 (31.3)  21 (16.0) | 27 (50.9)  2 (3.8)  11 (20.8)  22 (41.5)  3 (5.7) | 0.527  0.646  0.796  0.186  0.059 |
| **Tumor biomarkers****, n (%)**  Ca19-9 ≥ 37 U/mL  CEA > 5 ng/mL | 31 (23.7)  17 (13.0) | 10 (18.9)  5 (9.4) | 0.479  0.502 |
| **Pathologic grade, n (%)**  Low-grade dysplasia  Intermediate-grade dysplasia  High-grade dysplasia  Invasive carcinoma | 27 (20.6)  51 (38.9)  23 (17.6)  30 (22.9) | 16 (30.2)  16 (30.2)  10 (18.9)  11 (20.8) | 0.496 |
