# Supplementary material for: Circulating cytokines allow for identification of malignant intraductal papillary mucinous neoplasms of the pancreas
Source: Cancer Med. 2022 Jul 24;12(4):3919–30. doi: 10.1002/cam4.5051 (PMC9972143; doi:10.1002/cam4.5051)
Supplement: Supplementary file 5 — Table S2 [file CAM4-12-3919-s004.docx]

**Supplementary Table 2. Demographic and clinical characteristics of patients with malignant IPMN in the validation cohort.**

| **Variables** | **Benign IPMN**  **(n=32)** | **Malignant IPMN**  **(n=21)** | **P value** |
| --- | --- | --- | --- |
| **Age****, median (IQR)** | 65.5 (62.0-68.8) | 69.0 (60.0-72.0) | 0.321 |
| **Gender, n (%)**  Female  Male | 12 (37.5)  20 (62.5) | 12 (57.1)  9 (42.9) | 0.160 |
| **Clinical, n (%)**  Symptoms  Local compression  Upper abdominal pain  Jaundice  Pancreatitis history  Dyspepsia | 10 (31.3)  1 (3.1)  7 (21.9)  2 (6.3)  0  6 (18.8) | 10 (47.6)  0  7 (33.3)  0  2 (9.5)  4 (19) | 0.229  1.000  0.355  0.512  0.152  1.000 |
| **Surgery, n (%)**  cWhipple  Distal pancreatectomy spleen preserving  Distal pancreatectomy with splenectomy  Local resection  Total resection | 17 (53.1)  1 (3.1)  13 (40.6)  1 (3.1)  0 | 8 (38.1)  0  10 (47.6)  0  3 (14.3) | 0.105 |
| **Location, n (%)**  Head  Body  Tail  Overlaps | 18 (56.3)  12 (37.2)  2 (6.3)  0 | 9 (42.9)  6 (28.6)  5 (23.8)  1 (4.8) | 0.133 |
| **Subtype, n (%)**  Branch-duct IPMN  Main-duct IPMN  Mixed-type IPMN | 12 (37.5)  3 (9.4)  17 (53.1) | 6 (28.6)  7 (33.3)  8 (38.1) | 0.092 |
| **Cyst diameter (cm), median (IQR)** | 2.5 (1.5-3.5) | 3.0 (1.9-4.9) | 0.427 |
| **Main duct diameter (mm), median (IQR)** | 5.7 (3.3-7.3) | 6.9 (3.8-10.8) | 0.116 |
| **High-risk stigmata, n (%)**  Obstructive jaundice  Solid component  MPD dilation ≥ 10 mm | 1 (3.1)  3 (9.4)  2 (6.3) | 0  9 (42.9)  9 (42.9) | 1.000  **0.012**  **0.004** |
| **Worrisome features, n (%)**  Cyst size > 3 cm  Pancreatitis  Thickened enhancing cyst walls  MPD 5 -9 mm  Lymphadenopathy | 16 (50.0)  0  3 (9.4)  14 (43.8)  2 (6.3) | 11 (52.4)  2 (9.5)  8 (38.1)  10 (47.6)  1 (4.8) | 0.865  0.152  **0.030**  0.121  1.000 |
| **Tumor biomarkers****, n (%)**  Ca19-9 ≥37 U/mL  CEA> 5 ng/mL | 3 (9.4)  2 (6.3) | 7 (33.3)  3 (14.3) | 0.069  0.618 |
| **Circulating cytokines, median (IQR)**  TNF-α, pg/mL  IL-2R, U/mL  IL-6, pg/mL  IL-8, pg/mL | 7.5 (5.8-11.8)  341.5 (232.5-416.8)  3.5 (2.2-4.4)  16.5 (10.0-23.0) | 15.3 (8.6-43.5)  406 (289.0-621.0)  5.0 (3.3-9.5)  27.0 (19.0-59.5) | **0.001**  **0.030**  **0.011**  **0.007** |
| **Pathologic grade, n (%)**  Low-grade dysplasia  Intermediate-grade dysplasia  High-grade dysplasia  Invasive carcinoma | 16 (50.0)  16 (50.0)  -  - | -  -  10 (47.6)  11 (52.4) | - |
