# Supplementary material for: Circulating cytokines allow for identification of malignant intraductal papillary mucinous neoplasms of the pancreas
Source: Cancer Med. 2022 Jul 24;12(4):3919–30. doi: 10.1002/cam4.5051 (PMC9972143; doi:10.1002/cam4.5051)
Supplement: Supplementary file 6 — Table S3 [file CAM4-12-3919-s001.docx]

**Supplementary Table 3. Discriminatory performance of TNF-α, IL-2R, IL-6, IL-8 and their combined model for detecting patients with malignant IPMNs in the validation cohort.**

| **Variables** | **AUC (95% CI)** | **Sensitivity** | **Specificity** | **PPV** | **NPV** |
| --- | --- | --- | --- | --- | --- |
| **TNF-α** | 0.776 (0.652-0.900) | 0.571 | 0.875 | 0.750 | 0.757 |
| **IL-2R** | 0.677 (0.525-0.830) | 0.429 | 0.937 | 0.818 | 0.714 |
| **IL-6** | 0.709 (0.559-0.859) | 0.667 | 0.750 | 0.636 | 0.774 |
| **IL-8** | 0.719 (0.580-0.859) | 0.857 | 0.531 | 0.545 | 0.850 |
| **TNF-α&IL-2R&IL-6&IL-8** | 0.853 (0.748-0.957) | 0.905 | 0.687 | 0.655 | 0.917 |

AUC: area under the receiver-operating-characteristic curve; CI: confidence interval; PPV: positive predictive value; NPV: negative predictive value; Circulating cytokine score (TNF-α&IL-2R&IL-6&IL-8): Logit (P) = -6.439+0.109*TNF-α+0.008*IL-2R+0.35*IL-6+0.02*IL-8
