# Supplementary material for: Circulating cytokines allow for identification of malignant intraductal papillary mucinous neoplasms of the pancreas
Source: Cancer Med. 2022 Jul 24;12(4):3919–30. doi: 10.1002/cam4.5051 (PMC9972143; doi:10.1002/cam4.5051)
Supplement: Supplementary file 7 — Table S4 [file CAM4-12-3919-s006.docx]

**Supplementary Table 4. Independent risk factors associated with malignant IPMN by univariate and multivariate Logistic regression analysis in the validation cohort.**

| **Variables** | **Univariable P value** | **Multivariate P value** | **OR** | **95% CI** |
| --- | --- | --- | --- | --- |
| **Age (≥ 65 years)** | 0.938 | - |  |  |
| **Gender** | 0.163 | **-** |  |  |
| **Solid component** | **0.008** | **0.027** | 20.932 | 1.416-309.493 |
| **MPD dilation (≥ 10 mm)** | **0.004** | **0.004** | 76.417 | 3.839-1520.945 |
| **Thickened enhancing cyst walls** | **0.018** | **0.048** | 10.887 | 1.026-115.563 |
| **Ca19-9 (≥ 37 U/mL)** | **0.039** | **0.025** | 16.216 | 1.408-186.713 |
| **Circulating cytokine score** | **<0.001** | **0.010** | 20.188 | 2.053-198.497 |
